# Artificial intelligence in endoscopy and colonoscopy: a comprehensive bibliometric analysis of global research trends

**DOI:** 10.3389/fmed.2025.1532640

**Published:** 2025-05-30

**Authors:** Negin Letafatkar, Amr Ali Mohamed Abdelgawwad El-Sehrawy, KDV Prasad, Ahmad Alkhayyat, Ehsan Amini-Salehi, Maryam Hasanpour, Masoomeh Namdar Taleshani, Mohammad Hashemi, Hadi Alotaibi, Pegah Rashidian, Mohammad-Hossein Keivanlou, Soheil Hassanipour

**Affiliations:** ^1^Gastrointestinal and Liver Diseases Research Center, Guilan University of Medical Sciences, Rasht, Iran; ^2^Department of Internal Medicine, Diabetes, Endocrinology and Metabolism, Mansoura University, Mansoura, Egypt; ^3^Symbiosis Institute of Business Management, Hyderabad, India; ^4^Symbiosis International (Deemed University), Pune, India; ^5^Department of Computers Techniques Engineering, College of Technical Engineering, The Islamic University, Najaf, Iraq; ^6^Department of Computers Techniques Engineering, College of Technical Engineering, The Islamic University of Al Diwaniyah, Al Diwaniyah, Iraq; ^7^Department of Computers Techniques Engineering, College of Technical Engineering, The Islamic University of Babylon, Babylon, Iraq; ^8^Cardiovascular Research Center, Hormozgan University of Medical Sciences, Bandar Abbas, Iran; ^9^Department of Medicine, Vision Colleges, Riyadh, Saudi Arabia

**Keywords:** adenoma detection, artificial intelligence, bibliometric analysis, cancer, colonoscopy, diagnosis, endoscopy

## Abstract

**Background:**

Artificial intelligence (AI) has revolutionized the field of gastroenterology, particularly in endoscopic and colonoscopic procedures. These AI technologies aim to enhance diagnostic accuracy by facilitating the detection of gastrointestinal lesions, such as polyps and neoplasms. However, the rapid expansion of research in this area necessitates a comprehensive analysis to assess global trends and contributions. This study aims to conduct a thorough bibliometric and visualization analysis of global research focused on AI applications in endoscopy and colonoscopy.

**Methods:**

A systematic search was conducted in September 2024 using the Web of Science Core Collection. The data were analyzed using VOSviewer, CiteSpace, and R software, focusing on co-authorship, co-citation, and keyword trends.

**Results:**

Research output on AI in endoscopy and colonoscopy has seen significant growth since 2016, peaking in 2023 with 345 publications. The top contributing country was China, with 399 publications, while the United States led in centrality with a score of 0.27, indicating its key position in research collaborations. Showa University contributed the highest number of institutional publications (64 papers). Mori Y emerged as the leading author, with 53 publications, reflecting his significant influence in the field. The leading journal was Gastrointestinal Endoscopy, contributing 72 publications and accumulating 6,496 citations. The most frequently occurring keywords were “diagnosis,” “classification,” and “cancer.” The cluster analysis identified key research areas, with newer clusters emerging around “adenoma detection,” “polyp segmentation,” and “wireless capsule endoscopy.” These clusters have shown an increasing trend over the past few years, reflecting the growing focus on using AI to optimize diagnostic procedures in real-time.

**Conclusion:**

The bibliometric analysis highlights the rapid expansion and diversification of AI research in endoscopy and colonoscopy. Key clusters, such as “adenoma detection” and “polyp segmentation,” underscore the field's shift toward real-time diagnostic improvements. As AI technologies become more integrated into clinical practice, they are set to improve diagnostic accuracy and patient outcomes in gastroenterology.

## Introduction

The advent of artificial intelligence (AI) in medical diagnostics has markedly transformed the landscape of gastroenterology (1–4). AI technologies, encompassing machine learning and deep learning algorithms, have been increasingly integrated into these procedures to enhance the detection and characterization of gastrointestinal lesions (5–9). These innovations aim to augment diagnostic accuracy, facilitate the early identification of abnormalities such as polyps and neoplasms, and ultimately improve patient outcomes (10–14).

Endoscopic and colonoscopic examinations are pivotal for the diagnosis, surveillance, and management of gastrointestinal diseases (15–17). Despite their clinical importance, these procedures are inherently operator-dependent, leading to variability in detection rates and diagnostic accuracy (18, 19). The incorporation of AI serves to mitigate these limitations by providing real-time image analysis and decision support, thereby standardizing examinations and reducing the incidence of missed lesions (20, 21). Furthermore, AI applications have the potential to optimize procedural efficiency and reduce the cognitive load on clinicians (22).

Bibliometric studies offer a systematic approach to quantitatively evaluating scientific publications, enabling the assessment of research performance, identification of influential contributions, and mapping of collaborative networks within a specific domain (23–25). By employing bibliometric methods alongside visualization tools, complex datasets can be transformed into interpretable visual representations, facilitating a deeper understanding of research trends and knowledge structures. A comprehensive bibliometric analysis of AI applications in endoscopy and colonoscopy is particularly valuable because of the rapid expansion, interdisciplinary nature, and global interest surrounding AI-driven medical technologies. Such analysis systematically quantifies and visually maps research contributions, collaboration patterns, influential studies, and evolving thematic trends, which traditional literature reviews might not fully capture. By objectively analyzing publication trends, author networks, and thematic clusters, bibliometric studies can identify knowledge gaps, highlight emerging research directions, and provide researchers, clinicians, and policymakers with data-driven insights necessary for guiding future research investments, policy decisions, and clinical practice advancements in the rapidly evolving field of AI-enhanced gastroenterology. The objective of this study is to conduct a thorough bibliometric and visualization analysis of global research pertaining to AI applications in endoscopy and colonoscopy.

## Methods

### Data collection

**Figure 1:**
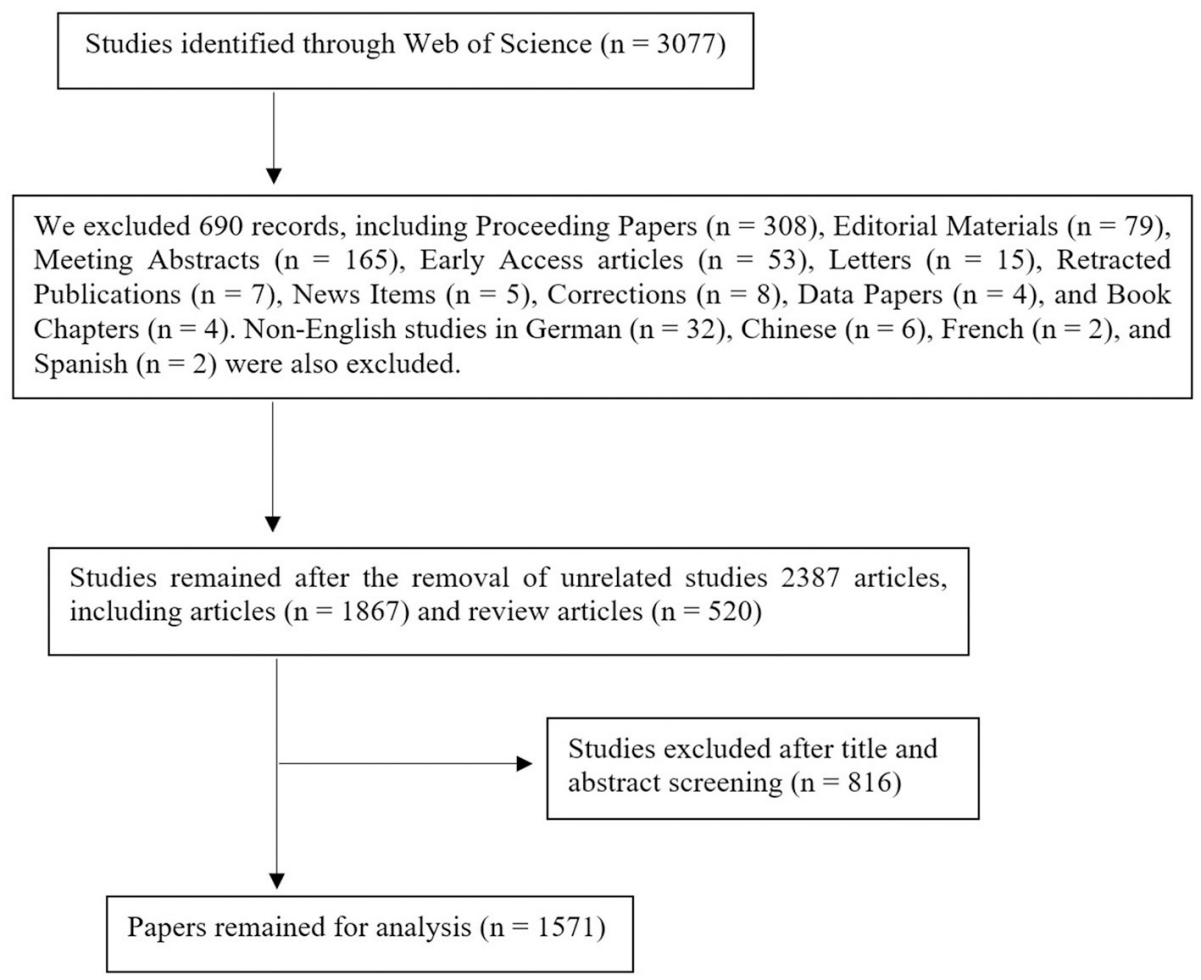
Study selection process.

A search was performed on September 5, 2024, within the Web of Science Core Collection, a well-regarded and extensive database that includes more than 12,000 respected publications, to obtain information on published articles (26–28). The inclusion criteria for selecting studies in this bibliometric analysis consisted of original research articles and review articles explicitly focused on AI applications in endoscopy and colonoscopy. Publications were excluded if they were not directly relevant to AI applications within this scope. Additionally, we excluded specific types of publications, including conference proceedings, editorials, book chapters, letters, retracted papers, non-English publications, and pre-publication articles. A variety of keywords, such as “Endoscopy,” “Capsule Endoscopy,” “Colonoscopy,” “Artificial Intelligence,” “Machine Learning,” and “Neural Network,” were utilized to develop a search strategy aimed at improving the effectiveness of the query (Supplementary Table S1). Initially, 3,077 papers were retrieved. Following the exclusion of book chapters, editorials, conference papers, letters, and pre-publication articles, a final set of 1,571 publications was selected (Figure 1).

The selection of only original research and review articles was driven by their thorough peer-review processes, which ensure the studies' credibility and scientific quality. Other forms of literature, such as conference proceedings, editorials, and books, were excluded since they generally do not undergo the same rigorous peer review or indexing, which could compromise the consistency and reliability of citation patterns in bibliometric analyses.

Additionally, retracted papers were removed to uphold the dataset's accuracy, as they no longer represent reliable scientific contributions. Non-English publications were also excluded because the bibliometric tools we utilized are designed for processing English-language text. Limiting the dataset to English papers helped maintain uniformity in the analysis, as these tools are not fully compatible with non-English content.

### Data screening

A detailed screening process was carried out to ensure the quality and relevance of the included studies. The titles, abstracts, and keywords of all retrieved publications were carefully examined. When relevance or quality could not be confirmed from these criteria, a full-text review was conducted. Through this approach, only studies specifically addressing AI applications in endoscopy and colonoscopy imaging were included.

### Data analysis

The documents downloaded from the Web of Science Core Collection were analyzed using VOSviewer (version 1.6.19), CiteSpace (version 6.4 R1), and Biblioshiny (version 4.0). The data were subsequently converted into CSV and plain text formats.

VOSviewer (accessible at www.vosviewer.com) is a well-established tool for constructing and visualizing bibliometric networks, helping researchers identify patterns in academic publication. It was developed by Nees Jan van Eck and Ludo Waltman at Leiden University's Center for Science and Technology Studies (29). VOSviewer specializes in mapping relationships such as co-authorship, co-citation, bibliographic coupling, and keyword co-occurrence.

Co-authorship analysis uncovers collaboration patterns between authors and institutions, providing insights into research partnerships (30). Co-citation analysis identifies papers or authors frequently cited together, revealing intellectual connections and prominent research communities (31). Bibliographic coupling groups papers that share common references, assisting in the discovery of related topics and emerging research fronts (32). Keyword co-occurrence analysis visualizes frequently used terms in publications, reflecting the thematic structure and evolving trends within a field (33).

VOSviewer uses text mining techniques, including sentence detection and part-of-speech tagging from the Apache OpenNLP library. Sentence detection divides text into individual sentences, while part-of-speech tagging assigns each word a part of speech (e.g., verb, noun, adjective), allowing for a more in-depth analysis of research themes. The software also employs distance-based visualization, where the proximity of nodes indicates the strength of their relationships, facilitating easier interpretation of complex bibliometric data (34–36).

CiteSpace, developed by Chaomei Chen at Drexel University (available at www.citespace.podia.com), is a specialized tool for visualizing and analyzing citation networks. It is particularly adept at detecting citation bursts and tracking the rise of emerging research trends. A citation burst signifies a rapid surge in the number of citations for a particular paper or topic, signaling its growing impact in the field (37).

CiteSpace excels in performing cluster analysis, where studies are grouped based on co-citation patterns, uncovering connections between different research areas. Clusters are labeled using log-likelihood ratio (LLR) tests, which automatically generate meaningful labels from key terms found in the cluster's articles. The quality of these clusters is assessed through modularity and silhouette scores. Modularity evaluates the internal structure of the network, with higher values indicating more distinct and loosely connected sub-networks. Silhouette scores measure the cohesion of clusters, with higher scores suggesting more consistent and meaningful groupings.

The software also offers time-slicing capabilities, enabling researchers to track the development of key concepts over specific periods. CiteSpace uses Kleinberg's burst detection algorithm to identify sudden spikes in citation activity, helping to highlight emerging trends or breakthroughs in research (38–40).

Biblioshiny (available at www.bibliometrix.org) is an intuitive graphical interface for the R Bibliometrix package, developed by Massimo Aria and Corrado Cuccurullo. It provides a robust set of tools for bibliometric analysis, enabling users to perform citation analysis to evaluate the impact of papers, authors, and institutions, as well as co-authorship analysis to explore collaboration patterns within networks. Moreover, Biblioshiny includes a variety of community detection algorithms, such as Louvain, Walktrap, and Multidimensional Scaling (MDS), which facilitate the identification of clusters in networks, helping to uncover related research fields or collaborative networks (41–44).

## Results

### Publication trend

**Figure 2:**
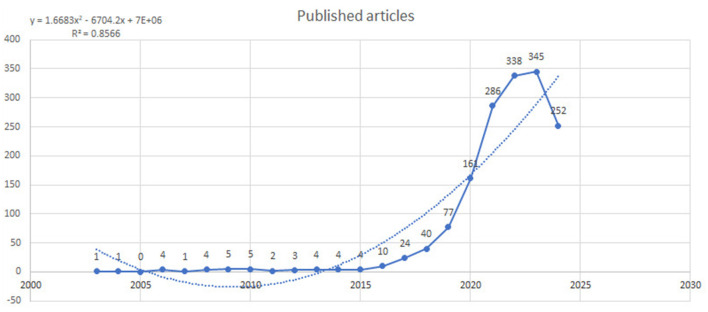
Annual number of published articles on artificial intelligence in endoscopy and colonoscopy from 1990 to 2024.

The trend analysis of research on AI in endoscopy and colonoscopy showed substantial growth in academic interest over the years. From 1993 to 2015, there was minimal research activity, with only a few articles published annually. During this period, the number of publications remained stagnant, with fewer than five articles per year. A notable increase in research output began in 2016, marking the start of an upward trend. The number of publications rose steadily, reaching a peak of 345 papers in 2023. This growth illustrates the rapidly increasing interest and focus on the application of AI technologies in endoscopy and colonoscopy. After peaking in 2023, there was a slight decline in 2024, with 252 publications, which could be attributed to the ongoing year and the typical delay in indexing articles (Figure 2).

**Figure 3:**
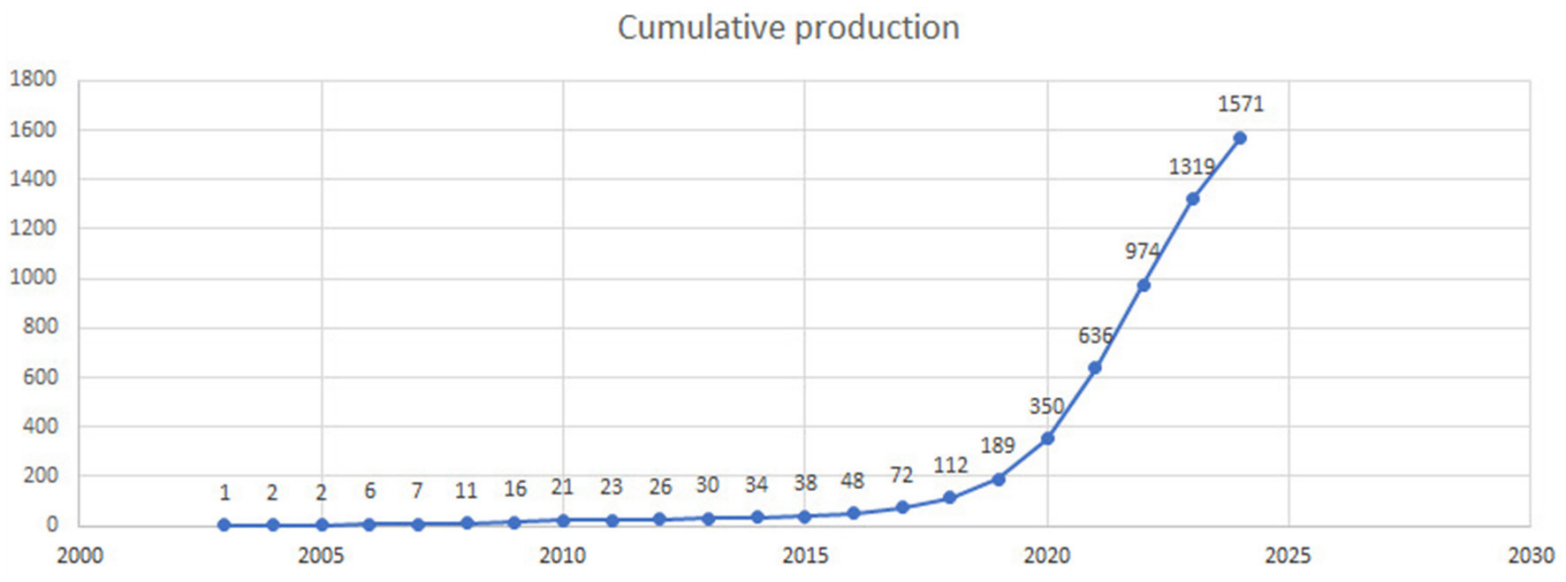
Cumulative number of publications on artificial intelligence in endoscopy and colonoscopy from 1990 to 2024.

The cumulative research output on AI in endoscopy and colonoscopy demonstrated a significant upward trajectory over the past few decades. From 1993 to 2019, the cumulative number of publications remained low. However, starting in 2020, the field experienced a steady increase, reaching 1,571 cumulative publications by 2024 (Figure 3).

### Countries and institutions

**Figure 4:**
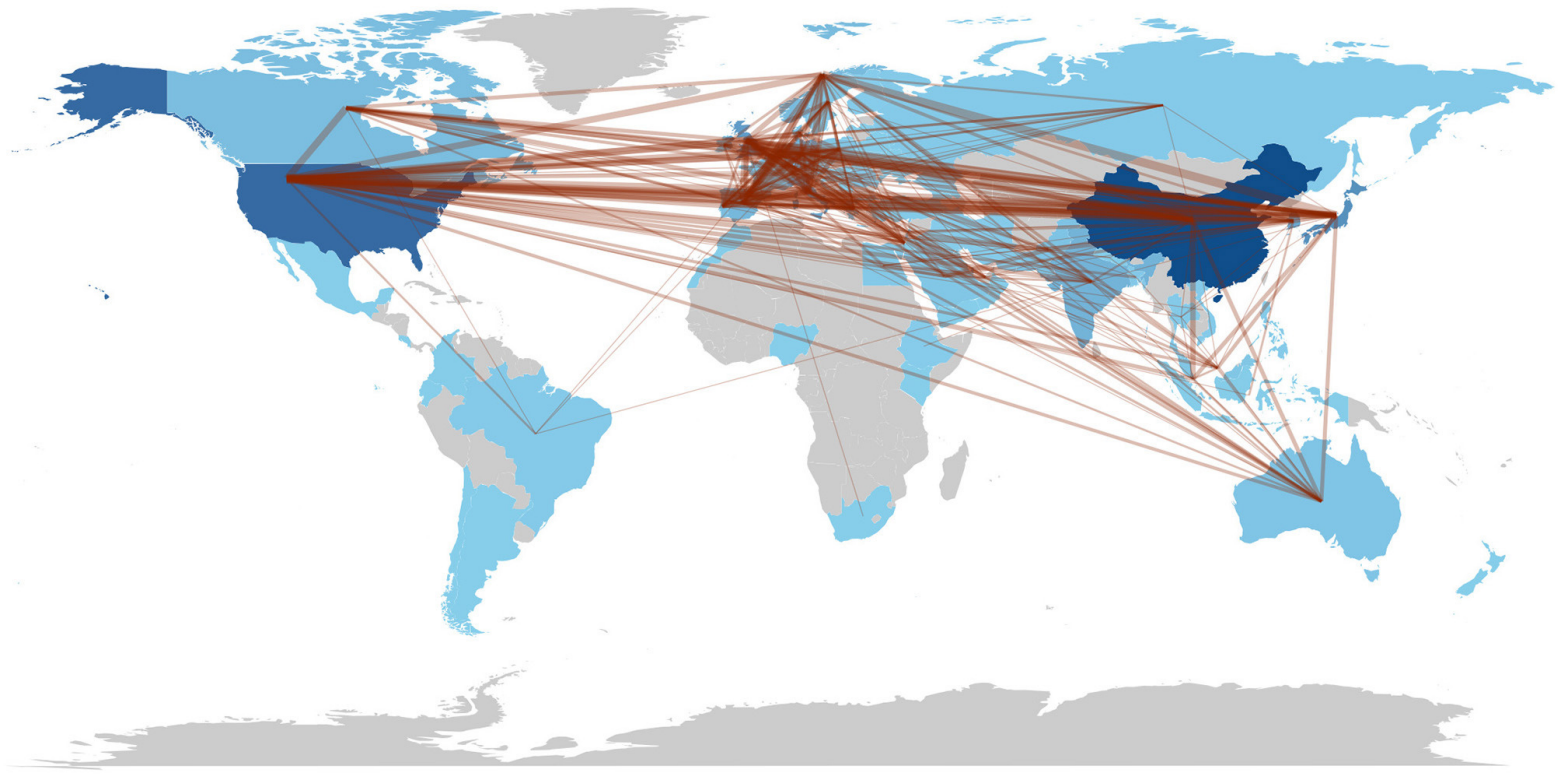
Countries collaboration regarding artificial intelligence in endoscopy and colonoscopy.

Figure 4 shows the global collaboration of the countries in the field. The top 10 countries that contributed to research on AI in endoscopy and colonoscopy, ranked by the number of publications, were as follows: the People's Republic of China led with 399 publications, followed by the United States with 334, and Japan with 202. England contributed 141 publications, Italy 132, and South Korea 121. Germany had 88 publications, India 86, Spain 68, and Norway rounded out the top 10 with 60 publications.

**Figure 5:**
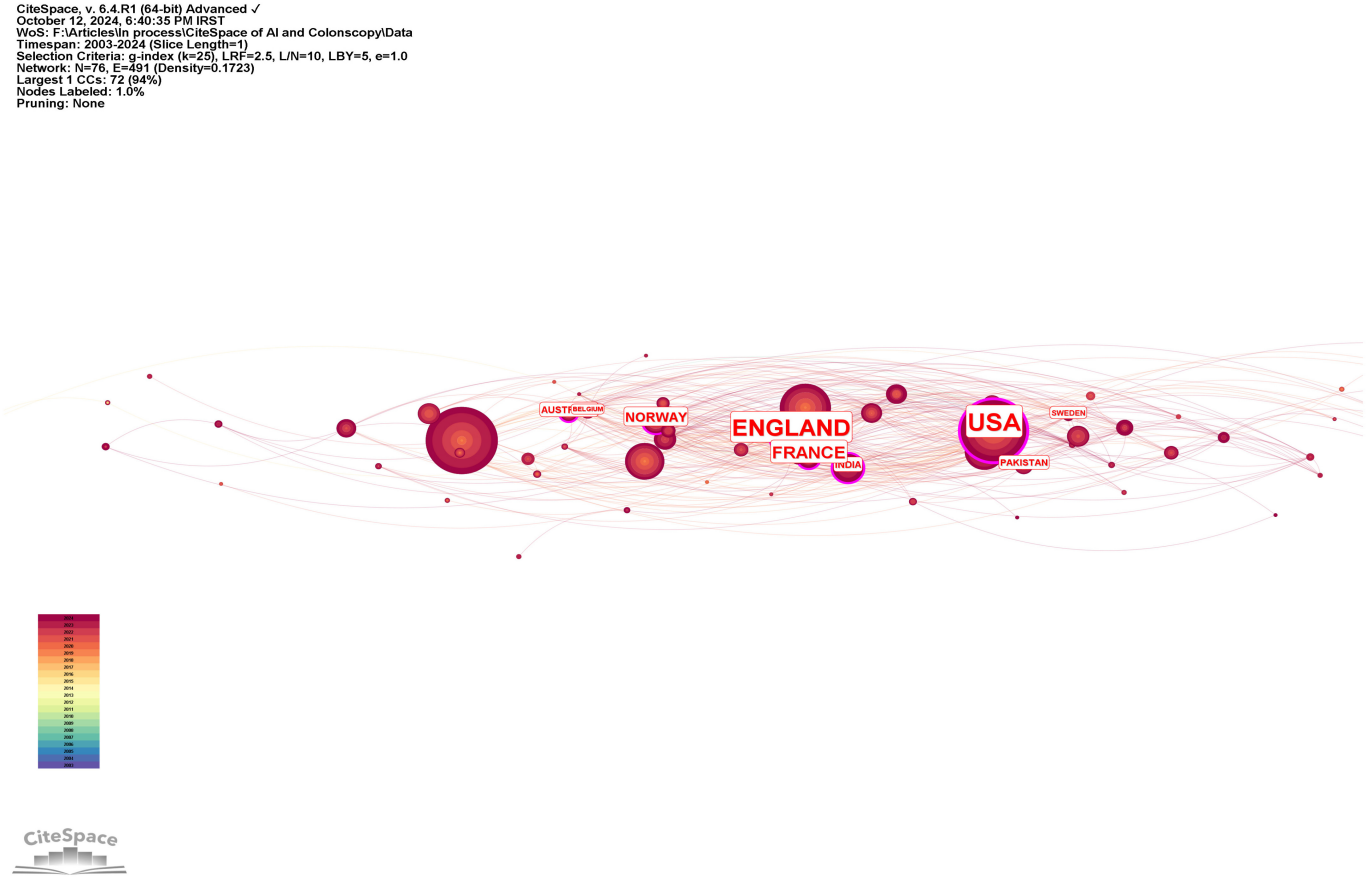
Leading countries regarding centrality in the field of artificial intelligence in endoscopy and colonoscopy.

**Figure 6:**
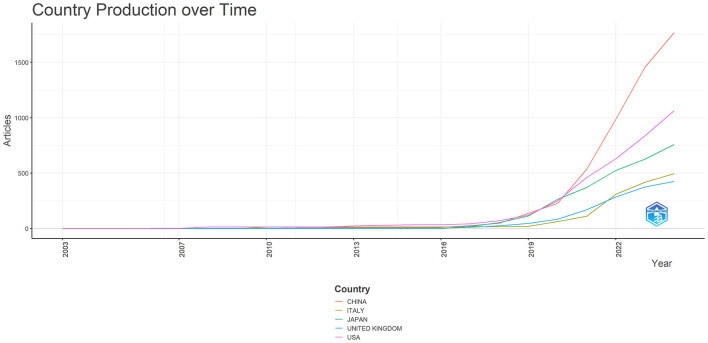
Countries production overtime in the field of artificial intelligence in endoscopy and colonoscopy.

**Figure 7:**
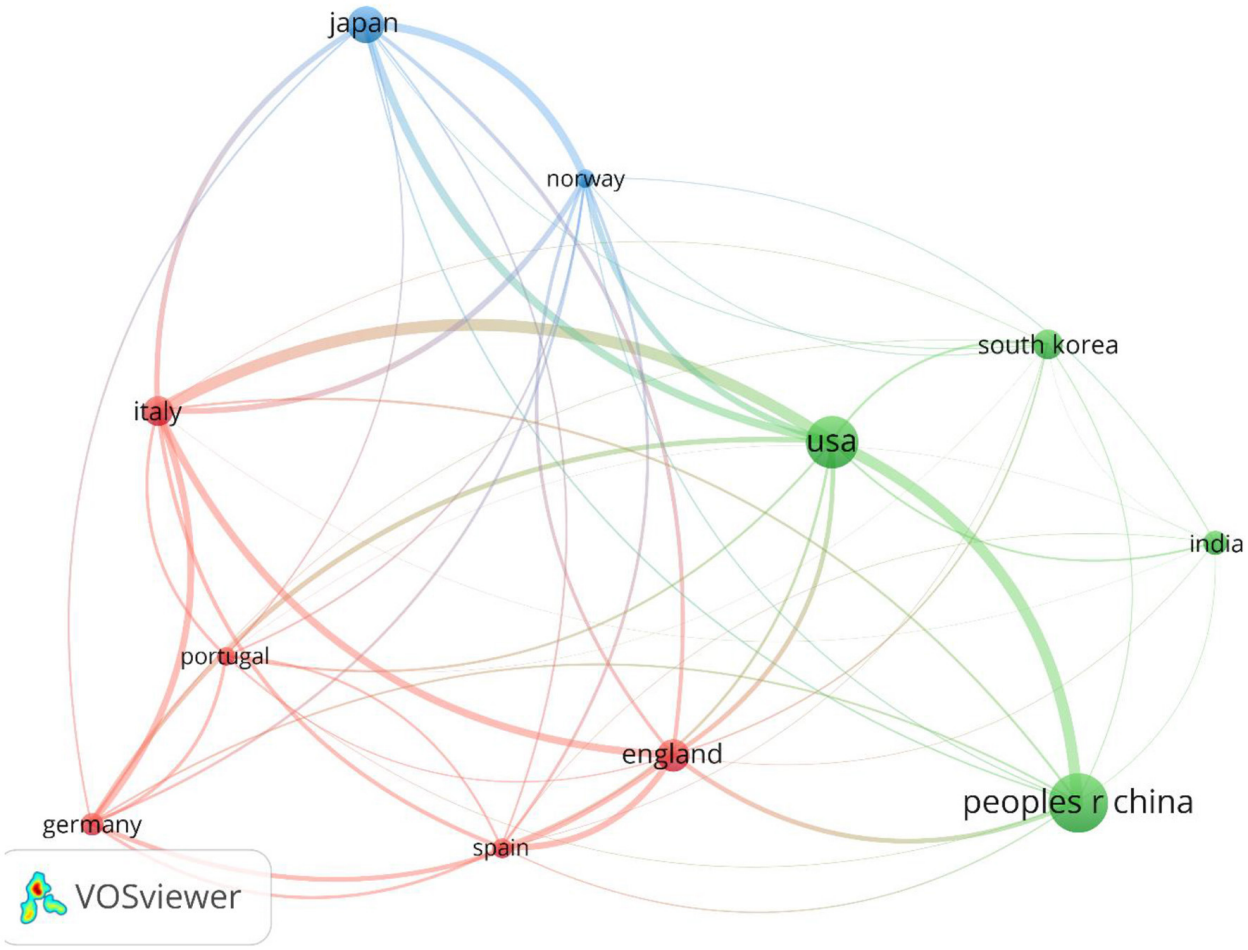
Network visualization of top countries in the field of artificial intelligence in endoscopy and colonoscopy.

The top 10 countries based on centrality in research on AI in endoscopy and colonoscopy were led by the United States with a centrality of 0.27, followed by England with 0.25. France had a centrality of 0.19, while Norway followed with 0.15. Australia, Pakistan, and India each had a centrality of 0.10, with Sweden at 0.08. Thailand and Saudi Arabia both had a centrality of 0.07, rounding out the top 10 (Figure 5). Figure 6 shows the countries' production over time. Figure 7 illustrates the relationship between the top countries in terms of publication and other countries.

Showa University topped the list of institutions contributing to research on AI in endoscopy and colonoscopy, with 64 publications. Humanitas University came next with 54, followed by the University of Oslo with 50. Harvard University released 48 publications, while the University of London contributed 45. Wuhan University added 44 publications to the field, and the University of Kansas published 37. Harvard Medical School produced 36 papers, while both the University of California System and University College London each contributed 35 publications.

**Figure 8:**
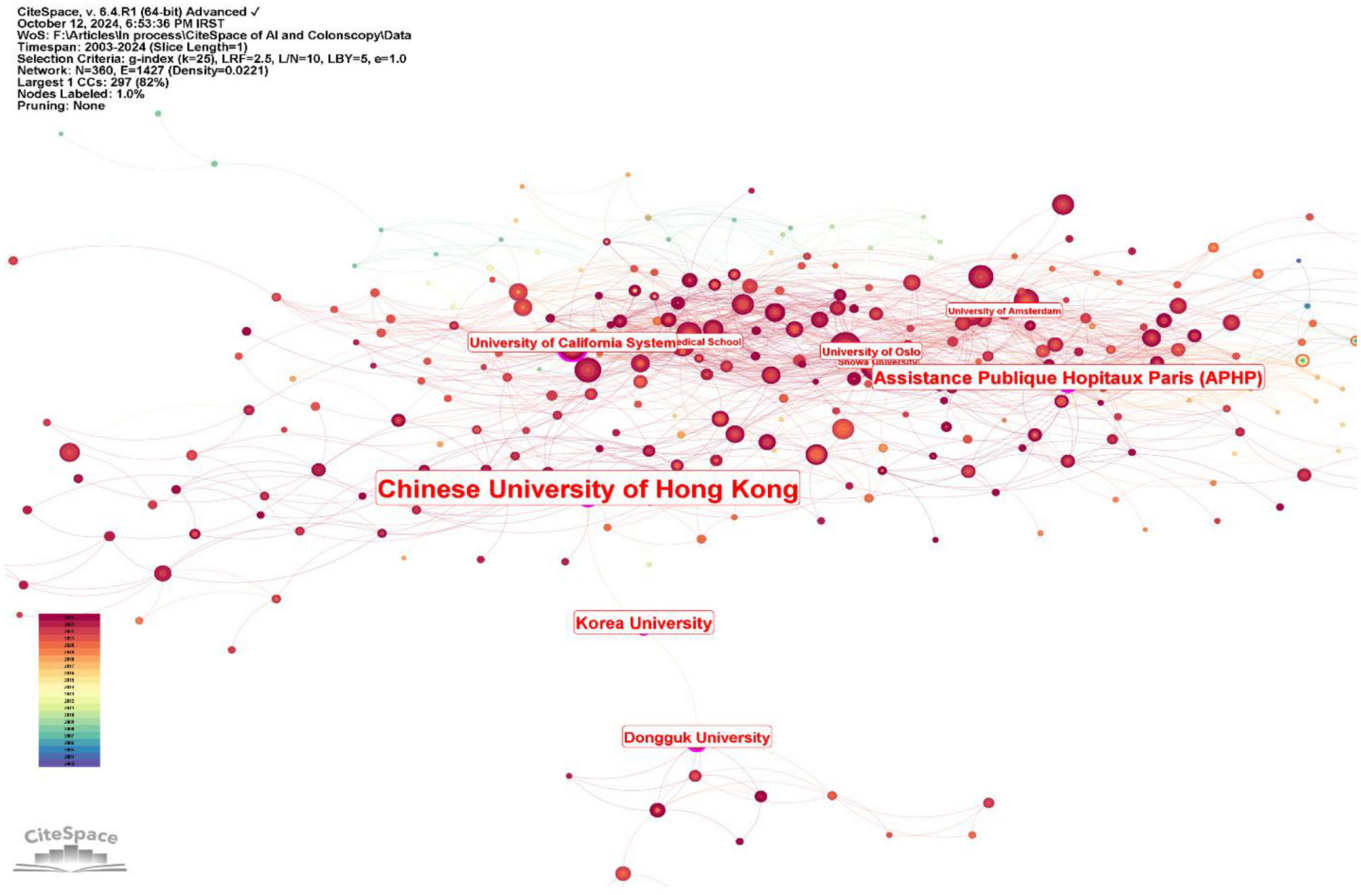
Leading institutions regarding centrality in the field of artificial intelligence in endoscopy and colonoscopy.

The top 10 institutions ranked by centrality in research on AI in endoscopy and colonoscopy were led by the Chinese University of Hong Kong, with a centrality score of 0.19. Assistance Publique Hopitaux Paris (APHP) followed with a score of 0.14, while Korea University achieved 0.12. Dongguk University and the University of California System had centrality scores of 0.11 and 0.10, respectively. The University of Oslo had a centrality of 0.09, followed by Showa University, Harvard Medical School, Indiana University Bloomington, and the University of Amsterdam, each with a score of 0.07 (Figure 8).

### Journals and co-cited journals

**Figure 9:**
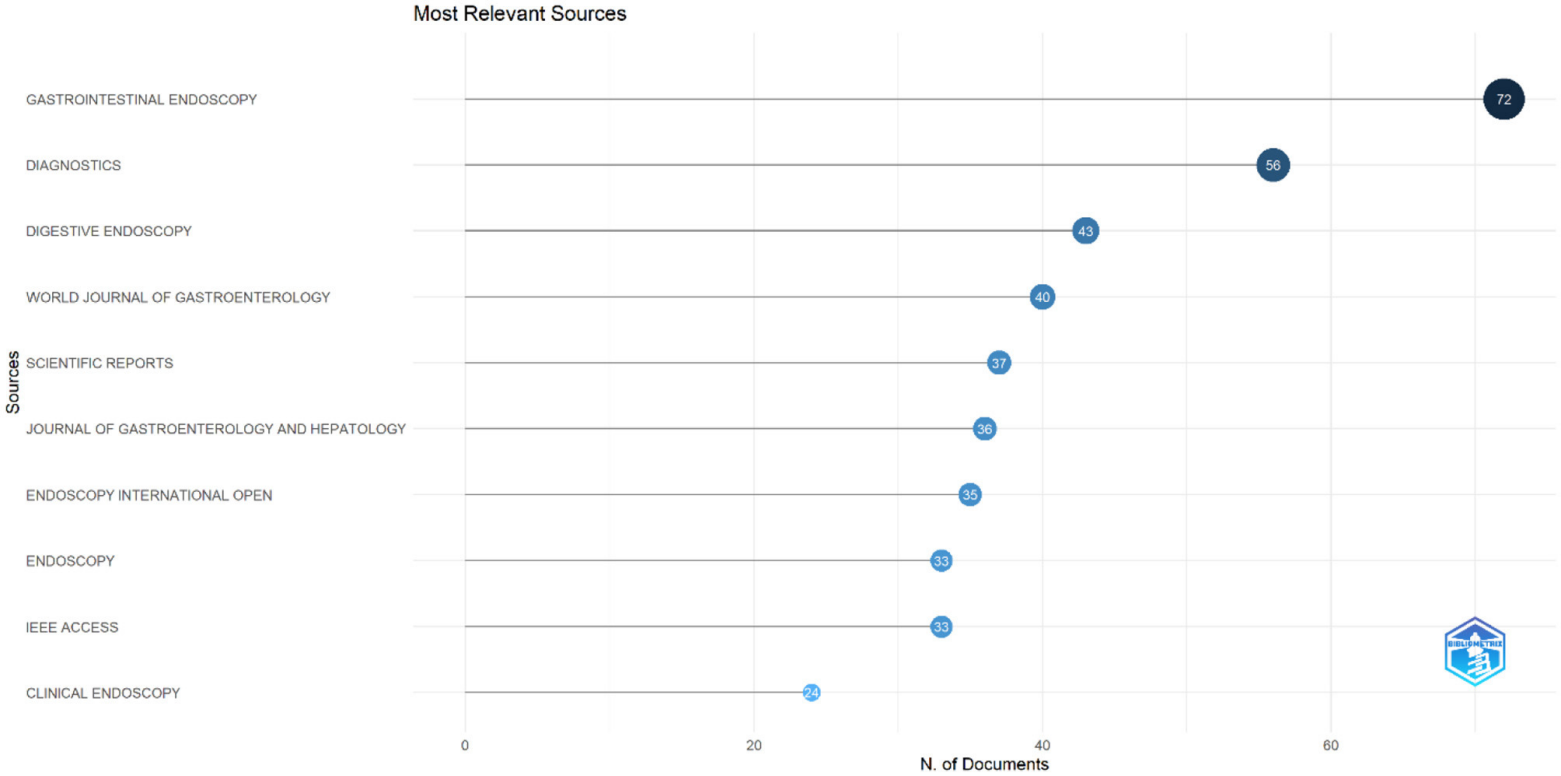
Leading journals in terms of publication in the field of artificial intelligence in endoscopy and colonoscopy.

The analysis identified the top 10 most relevant journals contributing to research on AI in endoscopy and colonoscopy. Gastrointestinal Endoscopy led the field with 72 publications, followed by Diagnostics with 56 publications. Digestive Endoscopy ranked third with 43 documents, while the World Journal of Gastroenterology contributed 40 publications. Other significant sources included Scientific Reports with 37 papers and the Journal of Gastroenterology and Hepatology with 36. Endoscopy International Open and Endoscopy each had 35 and 33 publications, respectively. IEEE Access also published 33 papers, and Clinical Endoscopy completed the top 10 with 24 publications (Figure 9).

**Figure 10:**
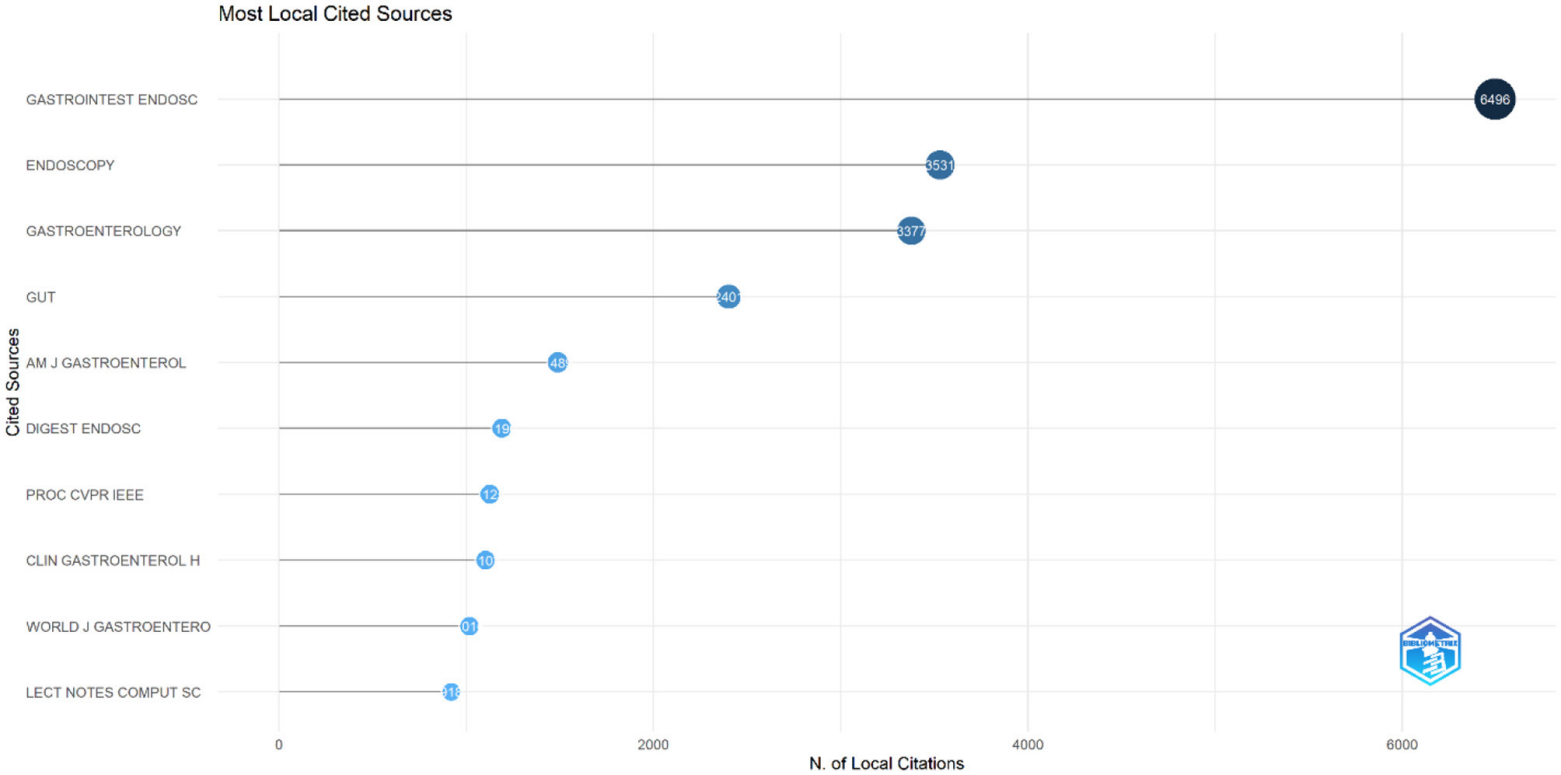
Leading cited journals in terms of publication in the field of artificial intelligence in endoscopy and colonoscopy.

**Figure 11:**
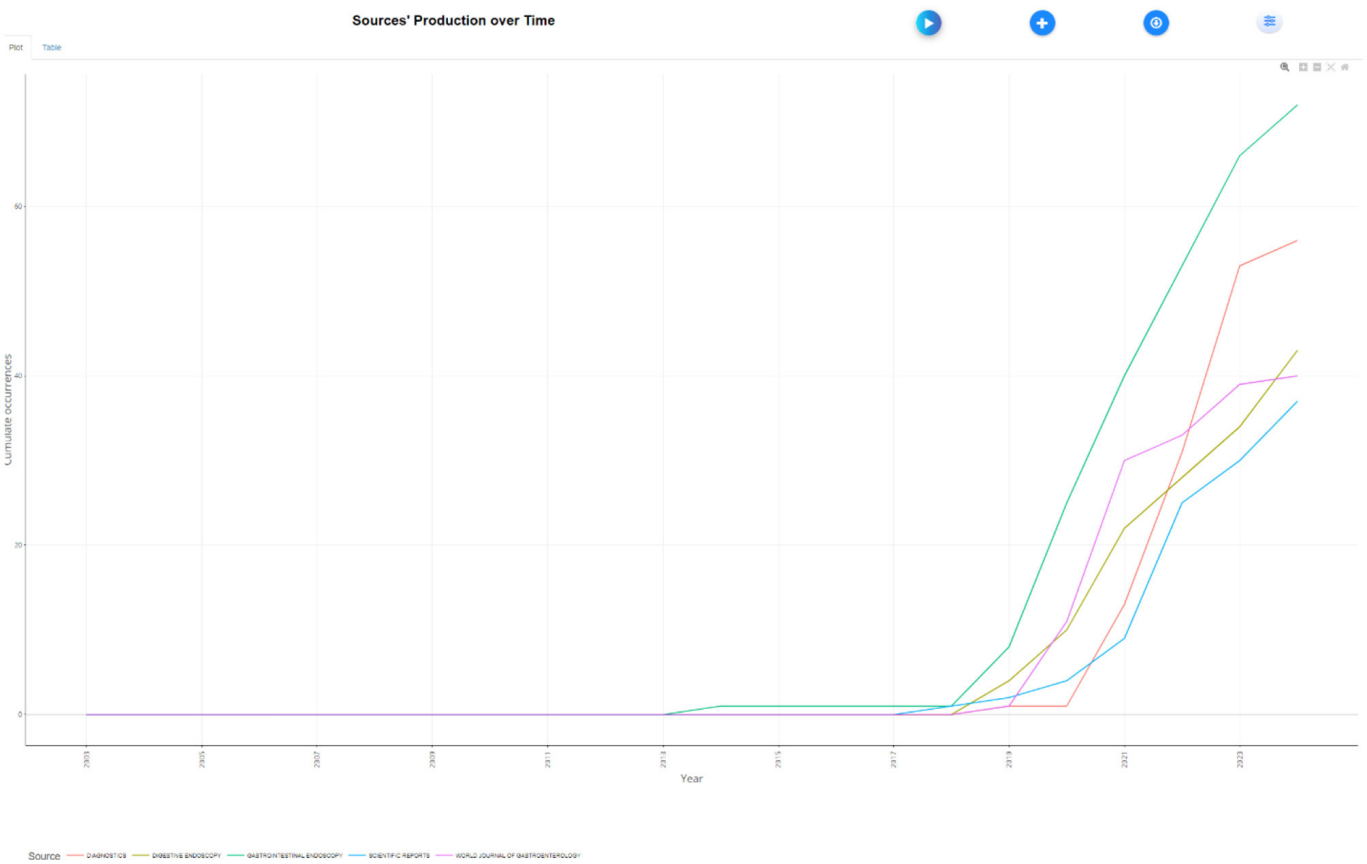
Journals' Productions over time in the field of artificial intelligence in endoscopy and colonoscopy.

The analysis of cited journals revealed that Gastrointestinal Endoscopy was the most frequently cited source, with 6,496 citations. Endoscopy followed closely with 3,531 citations, and Gastroenterology ranked third with 3,377 citations. Gut had 2,401 citations, while the American Journal of Gastroenterology received 1,489 citations. Other frequently cited journals included Digestive Endoscopy with 1,190 citations, Proceedings of CVPR IEEE with 1,225 citations, Clinical Gastroenterology and Hepatology with 1,102 citations, World Journal of Gastroenterology with 1,016 citations, and Lecture Notes in Computer Science with 918 citations (Figure 10). Figure 11 shows the journals' productions over time.

### Top cited papers

The bibliometric analysis identified the top 10 most-cited papers related to AI applications in endoscopy and colonoscopy. The most-cited paper was *Real-time automatic detection system increases colonoscopic polyp and adenoma detection rates: a prospective randomized controlled study*, published in 2018 in Gut, with 467 citations. The second most-cited paper was *Application of artificial intelligence using a convolutional neural network for detecting gastric cancer in endoscopic images*, published in 2018 in Gastric Cancer, with 465 citations. The third paper, *Deep Learning Localizes and Identifies Polyps in Real Time With 96% Accuracy in Screening Colonoscopy*, was published in 2018 in Gastroenterology and has 411 citations. The fourth paper was *Real-time differentiation of adenomatous and hyperplastic diminutive colorectal polyps during analysis of unaltered videos of standard colonoscopy using a deep learning model*, published in 2019 in Gut, with 392 citations. The fifth most-cited paper was *Real-Time Use of Artificial Intelligence in Identification of Diminutive Polyps During Colonoscopy: A Prospective Study*, published in 2018 in Annals of Internal Medicine, with 312 citations (Table 1).

**Table 1:** Top cited papers in the field of artificial intelligence in endoscopy and colonoscopy.

**Title**	**Published Year**	**Journal**	**Number of citations**
Real-time automatic detection system increases colonoscopic polyp and adenoma detection rates: a prospective randomized controlled study (62)	2018	Gut	467
Application of artificial intelligence using a convolutional neural network for detecting gastric cancer in endoscopic images (46)	2018	*Gastric Cancer*	465
Deep learning localizes and identifies polyps in real time with 96% accuracy in screening colonoscopy (47)	2018	Gastroenterology	411
Real-time differentiation of adenomatous and hyperplastic diminutive colorectal polyps during analysis of unaltered videos of standard colonoscopy using a deep learning model (48)	2019	Gut	392
Real-Time use of artificial intelligence in identification of diminutive polyps during colonoscopy: a prospective study (63)	2018	Annals of Internal Medicine	312
Efficacy of real-time computer-aided detection of colorectal neoplasia in a randomized trial (51)	2020	Gastroenterology	306
Functional interrogation and mining of natively paired human VH:VL antibody repertoires (45)	2018	Nature Biotechnology	290
Application of artificial intelligence to gastroenterology and hepatology (121)	2020	Gastroenterology	265
Accurate classification of diminutive colorectal polyps using computer-aided analysis (122)	2018	Gastroenterology	264
Performance of artificial intelligence in colonoscopy for adenoma and polyp detection: a systematic review and meta-analysis (49)	2021	Gastrointestinal Endoscopy	253

### Historiograph analysis

**Figure 12:**
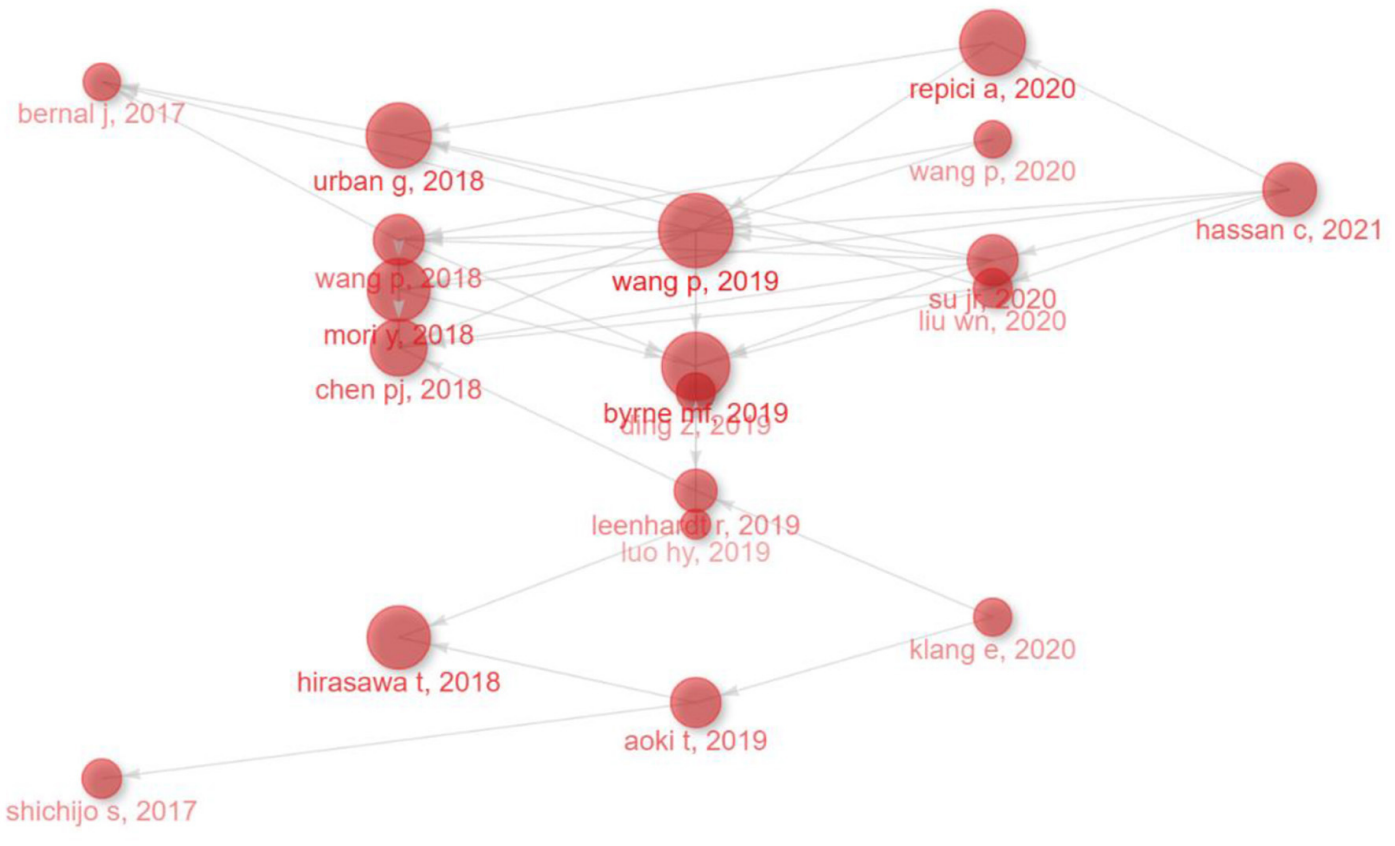
Historiograph analysis of influential publications in the field of artificial intelligence in endoscopy and colonoscopy.

To illustrate the evolution of research and identify influential milestone studies, a historiograph analysis was conducted. This analysis visually maps citation relationships between foundational and impactful studies in the field. The resulting historiograph (Figure 12) identified highly cited and interconnected articles, underscoring their significant influence on AI research in endoscopy and colonoscopy. In this map, each node represents a landmark publication, while the thickness of the connecting lines reflects the strength and frequency of citation relationships among these articles. The most prominent and influential studies identified by this analysis include: Wang et al. (45): “*Real-time automatic detection system increases colonoscopic polyp and adenoma detection rates: a prospective randomized controlled study,”* published in *Gut*. Hirasawa et al. (46): “*Application of artificial intelligence using a convolutional neural network for detecting gastric cancer in endoscopic images,”* published in *Gastric Cancer*. Urban et al. (47): “*Deep learning localizes and identifies polyps in real time with 96% accuracy in screening colonoscopy,”* published in *Gastroenterology*. Byrne et al. (48): “*Real-time differentiation of adenomatous and hyperplastic diminutive colorectal polyps during analysis of unaltered videos of standard colonoscopy using a deep learning model,”* published in *Gut*. Mori et al. (63): “*Real-Time Use of Artificial Intelligence in Identification of Diminutive Polyps During Colonoscopy: A Prospective Study,”* published in *Annals of Internal Medicine*.

The historiograph analysis also highlighted recent influential studies that significantly impacted AI research in endoscopy and colonoscopy. Among the most prominent recent nodes identified was the systematic review and meta-analysis by Hassan et al. (49), titled “*Performance of artificial intelligence in colonoscopy for adenoma and polyp detection,”* published in *Gastrointestinal Endoscopy*. Additionally, Wang et al. (50) conducted a study titled “*Lower Adenoma Miss Rate of Computer-Aided Detection-Assisted Colonoscopy vs Routine White-Light Colonoscopy in a Prospective Tandem Study,”* further evaluating AI's real-world clinical efficacy. Another influential study by Repici et al. (51), titled “*Efficacy of Real-Time Computer-Aided Detection of Colorectal Neoplasia in a Randomized Trial,”* published in *Gastroenterology*. These recent highly cited studies represent key advancements and have substantially impacted current research trends and clinical integration of AI in gastroenterology (Figure 12).

### Authors and co-cited authors

**Figure 13:**
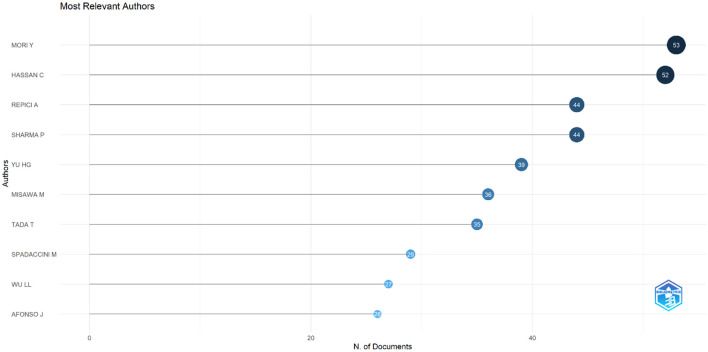
Top 10 authors regarding number of publications in the field of artificial intelligence in endoscopy and colonoscopy.

The top 10 authors contributing to research on AI in endoscopy and colonoscopy were led by Mori Y, with 53 publications. Hassan C followed closely with 52 publications. Repici A and Sharma P both contributed 44 publications each. Yu HG published 39 papers, while Misawa M had 36 publications. Tada T contributed 35 papers, followed by Spadaccini M with 29, Wu LL with 27, and Afonso J completing the list with 26 publications (Figure 13).

**Figure 14:**
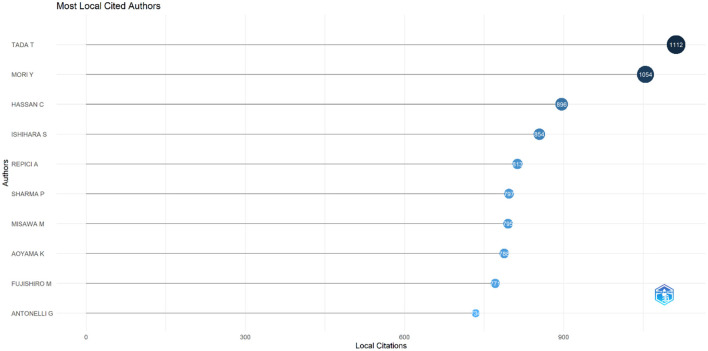
Top 10 most cited authors in the field of artificial intelligence in endoscopy and colonoscopy.

The top 10 most cited authors in research on AI in endoscopy and colonoscopy were led by Tada T, with 1,112 local citations. Mori Y followed closely with 1,054 citations, while Hassan C received 896 citations. Ishihara S garnered 854 citations, and Repici A accumulated 813 citations. Sharma P and Misawa M both had high citation counts, with 797 and 795 citations, respectively. Aoyama K had 788 citations, Fujishiro M followed with 771, and Antonelli G completed the top 10 with 734 citations (Figure 14). Supplementary Figure S1 shows the authors' productions over time.

### Key word trends, hotspots, cluster analysis

The most frequently occurring keywords in research on AI in endoscopy and colonoscopy were led by diagnosis, which appeared 276 times. Classification followed with 254 occurrences, while cancer was mentioned 201 times. Colonoscopy appeared 178 times, and system had 160 occurrences. Other commonly used keywords included artificial intelligence with 158 occurrences and validation with 156. Colorectal cancer and lesions each had 128 occurrences, while risk rounded out the top ten with 112 mentions (Supplementary Figure S2).

The analysis of keywords revealed that the most central terms in research on AI in endoscopy and colonoscopy were capsule endoscopy with a centrality score of 0.15, followed by algorithm with 0.12. Diagnosis had a centrality of 0.11, while computer-aided diagnosis had a score of 0.08. Other highly central keywords included classification, neural network, colorectal lesions, and colonic polyps, each with a centrality score of 0.07. Colorectal cancer, machine learning, and adenoma detection rate followed closely with a score of 0.06 each (Supplementary Figure S3).

The cluster analysis revealed the following major research areas: gastric cancer, polyp segmentation, wireless capsule endoscopy, adenoma detection, artificial intelligence, soft-computing methodologies, inflammatory bowel disease, regular colonoscopy, novel, and gastroenterology and hepatology (Supplementary Figure S4).

The time trend analysis of clusters revealed that certain research areas have gained attention in recent years. Notably, adenoma detection, polyp segmentation, wireless capsule endoscopy, and gastric cancer have seen an increase in research focus, with each cluster showing a rising trend in publications over the past few years (Supplementary Figure S5).

## Discussion

AI has experienced significant advancements since its inception, resulting in its integration into various medical disciplines. In endoscopy and colonoscopy, the adoption of AI began in the early 2000s, primarily to enhance diagnostic accuracy and operational efficiency (52, 53). Bibliometric analysis offers a systematic and quantitative approach to evaluating research trends by analyzing key metrics such as authorship, institutional contributions, country affiliations, and citation patterns. Unlike traditional systematic reviews, bibliometric methods, when combined with tools like CiteSpace and VOSviewer, provide a more rigorous and data-driven overview, offering visually interpretable insights into the evolving scientific landscape. This study represents the first comprehensive bibliometric analysis of AI applications in endoscopy and colonoscopy, covering the period from 2002 to 2023, with the aim of mapping the research trajectory and identifying emerging trends that could influence future developments in the field.

The publication trend analysis demonstrates a clear shift toward a growing academic focus on AI in endoscopy and colonoscopy. Initially, research activity in this area was limited, but over recent years, a notable increase in publications has emerged, signaling a substantial rise in interest. This trend reflects the growing momentum in the field, with more researchers contributing to the development and exploration of AI applications. The recent rise in cumulative publications further emphasizes this increasing engagement, suggesting that the field is gaining traction and becoming a prominent focus of scientific inquiry. The slight dip in recent publication numbers may reflect ongoing indexing processes rather than a reduction in research activity, highlighting the field's continued expansion.

The geographical distribution of research highlights the dominance of certain countries and institutions in driving innovation. An analysis of the countries and institutions contributing to research on AI in endoscopy and colonoscopy reveals that China holds the first rank in terms of publications, followed by the United States in second place and Japan in third place. In terms of institutional output, Showa University takes the top spot, followed by Humanitas University in second place and the University of Oslo in third place, reflecting their major contributions to advancing AI-driven diagnostic technologies.

Regarding centrality, which measures the influence and connectedness of a country or institution within the global research network, the United States ranks first, followed by England in second place and France in third place. Among institutions, the Chinese University of Hong Kong claims the top spot, with Assistance Publique Hopitaux Paris in second place and Korea University in third place, signifying their central roles in driving international collaboration and innovation in this field. Centrality highlights how pivotal these countries and institutions are in shaping global research directions and fostering important partnerships. The higher number of publications produced by China can be attributed to substantial government investment in AI research, rapid technological advancements, and significant growth in research institutions and researchers over recent years. In contrast, the leading role of the United States regarding collaboration centrality could stem from its well-established infrastructure for international research cooperation, active engagement in cross-country research initiatives, dedicated funding opportunities specifically targeting international collaboration, and a longstanding culture of collaborative scientific research. The higher number of publications produced by China can be attributed to substantial government investment in AI research, rapid technological advancements, and significant growth in research institutions and researchers over recent years. In contrast, the leading role of the United States regarding collaboration centrality could stem from its well-established infrastructure for international research cooperation, active engagement in cross-country research initiatives, dedicated funding opportunities specifically targeting international collaboration, and a longstanding culture of collaborative scientific research. Our bibliometric visualization analysis also highlighted thicker connecting lines between the United States and other leading countries, such as Japan, Germany, and England. These visually prominent connections underscore stronger international collaborative ties, further reinforcing the influential role of U.S. research outputs in shaping global research agendas and advancing the field of AI applications in endoscopy and colonoscopy.

The impacts of a higher number of publications include accelerated innovation, rapid dissemination of new AI technologies in clinical practice, and increased visibility of research institutions and individual researchers internationally (54–56). High publication output can facilitate faster knowledge accumulation, attracting further research funding and partnerships. Conversely, stronger global collaborations have distinct advantages. Robust international research collaborations often lead to higher-quality, multidisciplinary studies that benefit from diverse perspectives and expertise, thus enhancing scientific rigor and generalizability of results (57, 58). Collaborations can also expedite knowledge transfer, standardization of methodologies, and implementation of best practices across international boundaries, contributing significantly to shaping global research priorities and clinical guidelines in AI applications in endoscopy and colonoscopy (59–61). Our findings suggest that an ideal research environment would balance both high publication productivity and strong international collaboration, thereby maximizing both innovation and the practical impact of research outcomes.

The assessment of the most frequently cited references has revealed several key studies that have significantly contributed to the advancement of AI applications in endoscopy and colonoscopy. These cited papers are crucial in highlighting various aspects of AI which providing valuable insights into the development, clinical integration, and impact of AI technologies in gastrointestinal procedures.

The most cited study by Wang et al. (62) in *Gut 2019*, titled “Real-time automatic detection system increases colonoscopic polyp and adenoma detection rates: a prospective randomized controlled study,” explored the efficacy of an AI-based system for detecting polyps during colonoscopy. This randomized controlled trial found that the use of AI significantly improved adenoma detection rates (ADR) by 50%, increasing ADR from 20% to 30%. The study demonstrated that AI particularly aided in identifying smaller adenomas and hyperplastic polyps, contributing to enhanced diagnostic precision in real-time clinical settings. These findings underscore the potential of AI in improving colonoscopic outcomes, especially in detecting diminutive lesions.

The second most cited study by Hirasawa et al. (46) in Gastric Cancer 2018, titled “Application of artificial intelligence using a convolutional neural network (CNN) for detecting gastric cancer in endoscopic images,” focused on developing a CNN to automatically detect gastric cancer from endoscopic images. The study trained the AI model on over 13,000 images and tested it on a separate dataset. The CNN achieved a sensitivity of 92.2% in detecting gastric cancer lesions, particularly excelling in identifying larger lesions (≥6 mm) and invasive cancers. However, it missed smaller, superficially depressed lesions that were difficult even for experienced endoscopists to identify. Despite some false positives, the CNN demonstrated a strong potential for real-time clinical applications, reducing the diagnostic burden on endoscopists and improving detection rates for gastric cancer.

The third most cited study by Urban et al. (47) in Gastroenterology 2018, titled “Deep Learning Localizes and Identifies Polyps in Real Time with 96% Accuracy in Screening Colonoscopy,” developed a CNN to detect and localize polyps during colonoscopy in real-time. The CNN achieved a 96.4% accuracy and an area under the curve (AUC) of 0.991, identifying polyps with a high degree of precision. It was particularly effective in detecting polyps in real-time scenarios, reducing the risk of missed polyps during screenings. This study demonstrated the significant potential of AI-assisted systems to increase adenoma detection rates (ADR), which are critical in colorectal cancer prevention.

This fourth most cited study by Byrne et al. (48) in Gut 2019, titled “Real-time differentiation of adenomatous and hyperplastic diminutive colorectal polyps during analysis of unaltered videos of standard colonoscopy using a deep learning model,” developed a deep convolutional neural network (DCNN) to differentiate between adenomatous and hyperplastic diminutive polyps during colonoscopy. The AI model achieved an overall accuracy of 94%, with a sensitivity of 98% for identifying adenomas and a specificity of 83% for hyperplastic polyps. These results suggest that AI can provide real-time support in differentiating diminutive polyps, potentially facilitating the 'resect and discard' strategy, which could streamline clinical decision-making during colonoscopy and reduce unnecessary pathology evaluations.

This fifth most cited study by Mori et al. (63) in Annals of Internal Medicine 2018, titled “Real-Time Use of Artificial Intelligence in Identification of Diminutive Polyps During Colonoscopy: A Prospective Study,” focused on evaluating the performance of a computer-aided diagnosis (CAD) system during colonoscopy. The study, conducted on 791 patients, demonstrated that the CAD system achieved a negative predictive value (NPV) of 96.4% for diminutive rectosigmoid adenomas. This high NPV meets the clinical threshold required for a “diagnose-and-leave” strategy, reducing the need for unnecessary polyp resections. The findings suggest that CAD systems can effectively assist in real-time diagnosis of diminutive polyps, potentially improving the cost-effectiveness and efficiency of colonoscopy procedures.

The overall findings of these highly cited article indicate that research in this field has increasingly focused on using AI technologies to enhance the accuracy, efficiency, and real-time diagnostic capabilities in endoscopy and colonoscopy procedures. These studies highlight AI's role in improving the detection of malignancies, polyps, classification of diminutive polyps, and ultimately, the early identification of cancers such as colorectal and gastric cancer. Continuous advancements in AI-based diagnostic tools, including computer-aided diagnosis (CAD) systems and deep learning models, show a clear trend toward integrating AI into clinical practice. The goal is to reduce missed lesions, improve detection rates, and ultimately optimize patient outcomes.

An in-depth evaluation of key influential studies exemplifies the growing research emphasis on integrating artificial intelligence AI technologies to improve the diagnostic performance of colonoscopy. These studies have not only demonstrated the clinical value of AI-assisted approaches, but have also served as foundational milestones in shaping methodological standards and advancing the translational trajectory of AI in gastrointestinal endoscopy. Their collective contributions reflect a shift from early technical feasibility toward clinically meaningful, scalable, and evidence-based applications.

One of the most comprehensive and methodologically impactful studies in this field is the meta-analysis by Hassan et al., which pooled data from five randomized controlled trials with over 4,300 patients (49). This analysis provided robust evidence that AI-supported colonoscopy significantly improves ADR and adenomas per colonoscopy (APC), with consistent benefits across lesion size, morphology, and location. Importantly, it highlighted that AI performance remains effective regardless of lesion characteristics typically associated with higher miss rates, such as flat morphology, diminutive size, and proximal location. By emphasizing AI's independence from conventional perceptual constraints, this study underscored the capability of algorithm-driven systems to standardize detection quality and reduce observer variability in routine clinical practice.

Similarly, Wang et al. conducted one of the first prospective tandem colonoscopy trials to directly measure AI's impact on adenoma miss rate (AMR) (50). Their study demonstrated a substantial reduction in AMR—from 40.0% in standard procedures to 13.9% with AI support—particularly in the proximal colon. Beyond its clinical findings, this study introduced a rigorous methodological framework for evaluating recognition-related errors and positioned AI as a real-time adjunct capable of supporting endoscopists across varying levels of expertise. It also contributed significantly to the understanding of how AI can mitigate human cognitive limitations without disrupting procedural flow.

Repici et al. expanded the evidence base by implementing a multicenter randomized trial in a Western population, confirming AI's generalizability and feasibility in diverse clinical settings (51). Their findings demonstrated a marked increase in ADR (54.8% vs. 40.4%) and improved APC, with no significant difference in withdrawal time or unnecessary resections. The strength of this study lies in its pragmatic design and its demonstration that AI-enhanced colonoscopy can be seamlessly incorporated into existing endoscopic workflows. In particular, its effectiveness in detecting small and non-polypoid lesions further validates the role of AI in elevating detection quality in real-world practice.

Together, these milestone studies have shaped the evidence landscape by demonstrating not only the diagnostic advantages of AI, but also the importance of robust study designs, interdisciplinary collaboration, and clinical relevance in advancing the field. They collectively represent a transition point from proof-of-concept technologies to validated, practice-ready tools.

The analysis of publication trends underscores the prominent role of a few key journals in advancing AI research in endoscopy and colonoscopy. Gastrointestinal Endoscopy leads as the primary hub for cutting-edge AI research, emphasizing its crucial role in integrating AI into clinical practice. Diagnostics follows, reflecting the growing focus on AI's impact on diagnostic improvements, while Digestive Endoscopy, ranked third, highlights its importance in AI-driven real-time imaging and lesion detection. In terms of influence, Gastrointestinal Endoscopy also holds the top spot for citations, with foundational AI studies frequently referenced. Endoscopy and Gastroenterology follow closely, reinforcing their roles in promoting high-impact AI research and accelerating the adoption of AI technologies in gastroenterology.

In the keyword analysis of AI research in endoscopy and colonoscopy, diagnosis emerged as the most frequently mentioned term, reflecting the field's strong focus on enhancing the early detection of gastrointestinal lesions, particularly polyps and tumors. AI technologies significantly improve diagnostic accuracy, leading to better patient outcomes by facilitating timely interventions (64–66). Cancer, is another critical area of focus due to its global prevalence. AI plays a pivotal role in early detection during colonoscopy, reducing missed diagnoses, and improving overall screening efficiency (67–69). The frequent mention of classification further highlights AI's crucial role in distinguishing between benign and malignant lesions. AI-powered classification systems, driven by machine learning and deep learning algorithms, greatly enhance the precision and reliability of diagnostic assessments in clinical practice (70–72).

In the analysis of central keywords, particular emphasis is placed on diagnosis, which is further strengthened by AI-driven classification systems (73, 74). Innovations such as capsule endoscopy enable non-invasive, precise detection of gastrointestinal abnormalities, allowing AI to perform real-time classification of lesions (75–77). Keywords related to cancer and malignant conditions, such as colorectal cancer, colorectal lesions, adenoma detection rate, and colonic polyps, were frequently identified in the analysis. These terms highlight AI's critical role in detecting and classifying high-risk lesions (78, 79). AI technologies are especially effective in distinguishing malignant from benign polyps, improving the accuracy of early diagnosis and supporting clinicians in making more informed treatment decisions (80, 81). Although our analysis included 1,571 studies, the explicit keyword “Artificial Intelligence” appeared with a frequency of 158 occurrences (approximately 4%). It is crucial to emphasize that this frequency reflects only explicit occurrences of the term “Artificial Intelligence” itself, rather than indicating the number of studies using AI. The analyzed literature included numerous related keywords, some directly describing AI techniques (e.g., “neural network,” “classification,” “machine learning”), others describing AI's diagnostic applications (e.g., “diagnosis,” “validation”), and others specifically focused on gastrointestinal terminology (e.g., “endoscopy,” “lesion,” “cancer”). This diversity in terminology explains the relatively lower frequency of the specific phrase “Artificial Intelligence,” despite all analyzed studies being directly relevant to AI applications in endoscopy and colonoscopy.

The cluster analysis highlights four key research areas where AI is making significant contributions in endoscopy and colonoscopy. Gastric cancer emerges as a major focus, with AI technologies enhancing early detection and diagnosis, which is crucial for improving treatment outcomes (82–84). Polyp segmentation is another critical area, where AI automates the identification and delineation of polyps, helping to standardize detection and reduce missed polyps during colonoscopy, a vital step in preventing colorectal cancer (85–87). In wireless capsule endoscopy, AI optimizes the analysis of vast image datasets, improving diagnostic accuracy while reducing the workload for clinicians (88). Lastly, adenoma detection benefits from AI's ability to increase detection rates of these precancerous polyps, significantly enhancing screening outcomes and reducing the risk of colorectal cancer (89–91). Similar to previous results, these areas emphasize the impact of AI on diagnosis, cancer detection, and AI-assisted classification in various types of malignancies.

The time trend analysis of clusters reveals that research in adenoma detection, polyp segmentation, wireless capsule endoscopy, and gastric cancer has gained significant momentum in recent years. This growing focus highlights the increasing importance of AI in enhancing diagnostic precision and early detection in gastrointestinal procedures. The rising trend in adenoma detection reflects AI's role in improving the identification of precancerous polyps, a key factor in preventing colorectal cancer (92–94). Polyp segmentation has also seen increased attention, as AI-driven models enhance the accuracy of polyp detection and reduce variability across clinicians (95, 96). Similarly, wireless capsule endoscopy is benefiting from AI's ability to analyze large image datasets efficiently, contributing to non-invasive diagnostic improvements (97, 98). Lastly, the surge in research on gastric cancer demonstrates the field's focus on leveraging AI for early diagnosis, which is critical for improving survival rates (99–101). These trends indicate a broad recognition of AI's potential to revolutionize gastrointestinal diagnostics, particularly in detecting and classifying various types of malignancies.

While AI technologies offer transformative potential for enhancing endoscopy and colonoscopy, their global clinical implementation faces challenges and ethical considerations (102, 103). A critical challenge involves standardizing AI algorithms across diverse populations, medical practices, and healthcare settings (104). Differences in patient demographics, disease prevalence, imaging equipment, and clinical workflows can lead to performance variability of AI models when applied outside their training environments (105–108). Ensuring AI generalizability requires extensive validation in multicentric and diverse cohorts worldwide.

Furthermore, ethical concerns such as algorithm transparency, accountability, patient privacy, and data security pose considerable obstacles (109–112). The “black-box” nature of AI algorithms raises important questions regarding transparency, potentially leading to mistrust among clinicians and patients (113–115). Establishing accountability for AI-driven clinical decisions remains complex and currently lacks clear international standards or regulatory guidance.

Additionally, the widespread clinical integration of AI raises potential issues of equity and fairness (116, 117). Disparities in access to advanced AI technologies, especially in low- and middle-income countries, could exacerbate existing global healthcare inequalities (118–120). Addressing these concerns requires international collaborative efforts to develop clear ethical frameworks, rigorous clinical validation standards, transparent reporting guidelines, and equitable technology dissemination strategies.

## Limitations and future suggestions

While this study offers a thorough bibliometric analysis of AI applications in endoscopy and colonoscopy, several limitations should be noted. First, the analysis was confined to articles indexed in the Web of Science Core Collection. Although this is a comprehensive resource, it may not cover relevant research from other databases. Additionally, only English-language publications were considered, which may have overlooked significant research in other languages, introducing a geographic bias that underrepresents contributions from non-English-speaking regions. Future research could leverage large language models (LLMs) to incorporate and examine non-English literature. Employing LLMs would enhance the depth of bibliometric studies, enabling the identification of nascent research clusters and offering a fuller representation of worldwide contributions to AI in endoscopy and colonoscopy.

Another limitation concerns citation bias. Bibliometric analysis heavily relies on citation counts, which can be affected by factors such as the reputation of journals, the timing of publication, and practices like self-citation. Therefore, citation-based metrics may not entirely capture the true influence of specific papers or authors. Moreover, given the rapidly advancing nature of AI research, newer studies may not have gathered substantial citations yet, potentially underrepresenting emerging research trends. Another important consideration, not extensively covered in our current analysis, involves the practical application and limitations of AI technologies in clinical practice worldwide or on a country-specific basis. Although our study provided a comprehensive overview of global research trends, future analyses could specifically examine variations in clinical adoption, practical challenges, regulatory environments, ethical considerations, cost-effectiveness, and healthcare infrastructure that influence how AI is implemented differently across countries. Such studies would be invaluable for understanding the real-world impact, identifying barriers to clinical integration, and guiding targeted policy decisions and resource allocation for broader and more equitable implementation of AI-driven endoscopy and colonoscopy practices globally. Finally, our analysis only includes papers published up to September 2024, meaning further developments in the field may not be reflected. The reported slight decline in publications observed in 2024 should be interpreted cautiously. Since the dataset was analyzed up to September 2024, the data for 2024 represents only a partial year, potentially leading readers to inaccurately conclude that research activity in AI applications for endoscopy and colonoscopy is decreasing. Therefore, this trend may simply reflect incomplete data rather than an actual reduction in research output.

## Conclusion

This bibliometric analysis provides insights into the rapidly growing field of AI applications in endoscopy and colonoscopy. The analysis highlights key contributors, such as China and the United States, with the latter leading in global research collaborations. Institutions like Showa University and influential authors such as Mori Y have emerged as pivotal in advancing the field. Leading journals, particularly Gastrointestinal Endoscopy, play a critical role in disseminating cutting-edge research.

The cluster analysis revealed emerging trends focused on critical areas such as adenoma detection, polyp segmentation, and wireless capsule endoscopy, reflecting the increasing clinical emphasis on enhancing real-time diagnostic capabilities. The most frequently used keywords—“diagnosis,” “classification,” and “cancer”—underscore the central role of AI in improving the accuracy and efficiency of endoscopic procedures. As AI technologies become more integrated into clinical practice, they are anticipated to significantly improve diagnostic accuracy by providing real-time image analysis, reducing inter-operator variability, and enhancing the detection rates of critical gastrointestinal conditions, such as colorectal cancer and adenomas. These advancements are expected to lead to earlier and more precise diagnoses, reduce missed lesions, and ultimately improve patient outcomes by facilitating timely and effective treatment interventions in gastroenterology.

## Data Availability

The data analyzed in this study is subject to the following licenses/restrictions: the data used for the analysis can be provided on a reasonable request from the corresponding author. Requests to access these datasets should be directed to ehsanaminisalehi1998@gmail.com.
