# Supplementary material for: Artificial intelligence in endoscopy and colonoscopy: a comprehensive bibliometric analysis of global research trends
Source: Front Med (Lausanne). 2025 May 30;12:1532640. doi: 10.3389/fmed.2025.1532640 (PMC12162488; doi:10.3389/fmed.2025.1532640)
Supplement: Supplementary file 1 [file Data_Sheet_1.docx]

**Table S1**: Search strategy and the key words utilized

| Database | Search formula |
| --- | --- |
| Web of Science | (("Endoscopy Surgical Procedures" OR "Endoscopic Procedure" OR "Endoscopic Surgical Procedures" OR "Endoscopic Surgical" OR "Endoscopic Surgical Procedure" OR "Endoscopic Surgical Procedures" OR "Endoscopy" OR "Surgical Endoscopy" OR "Surgical Procedure" OR "Endoscopic Endoscopy" OR "Gastroscopy" OR "Colonoscopy" OR "Capsule Endoscopy" OR "Colonoscopies" OR "Colonoscopic Surgical Procedures" OR "Colonoscopic Surgical Procedure" OR "Procedure, Colonoscopic Surgical" OR "Procedures, Colonoscopic Surgical" OR "Surgical Procedure, Colonoscopic" OR "Colonoscopic Surgery" OR "Colonoscopic Surgeries" OR "Surgeries, Colonoscopic" OR "Surgery, Colonoscopic" OR "Surgical Procedures, Colonoscopic") AND ("Artificial Intelligence" OR "Intelligence, Artificial" OR "Computer Reasoning" OR "Reasoning, Computer" OR "AI (Artificial Intelligence)" OR "Machine Intelligence" OR "Intelligence, Machine" OR "Computational Intelligence" OR "Intelligence, Computational" OR "Computer Vision Systems" OR "Computer Vision System" OR "System, Computer Vision" OR "Systems, Computer Vision" OR "Vision System, Computer" OR "Vision Systems, Computer" OR "Knowledge Acquisition (Computer)" OR "Acquisition, Knowledge (Computer)" OR "Knowledge Representation (Computer)" OR "Knowledge Representations (Computer)" OR "Representation, Knowledge (Computer)" OR "Machine Learning" OR "Neural Networks, Computer" OR "Computer Neural Network" OR "Computer Neural Networks" OR "Network, Computer Neural" OR "Networks, Computer Neural" OR "Neural Network, Computer" OR "Models, Neural Network" OR "Model, Neural Network" OR "Network Model, Neural" OR "Network Models, Neural" OR "Neural Network Model" OR "Neural Network Models" OR "Computational Neural Network" OR "Network, Computational Neural" OR "Networks, Computational Neural" OR "Neural Network, Computational" OR "Neural Networks, Computational" OR "Neural Network" OR "Neural Networks (Computer)" OR "Network, Neural (Computer)" OR "Networks, Neural (Computer)" OR "Neural Network (Computer)" OR "prediction model")) |

**
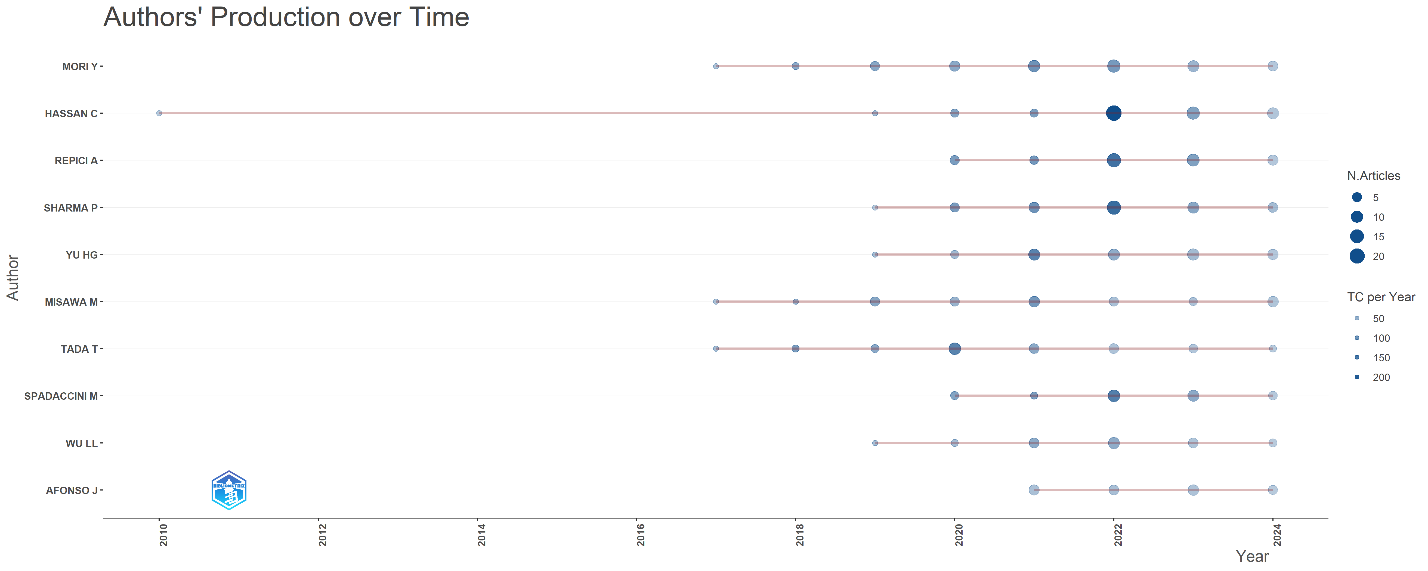
**

**Figure S1**: Authors’ Productions over time in the field of artificial intelligence in endoscopy and colonoscopy


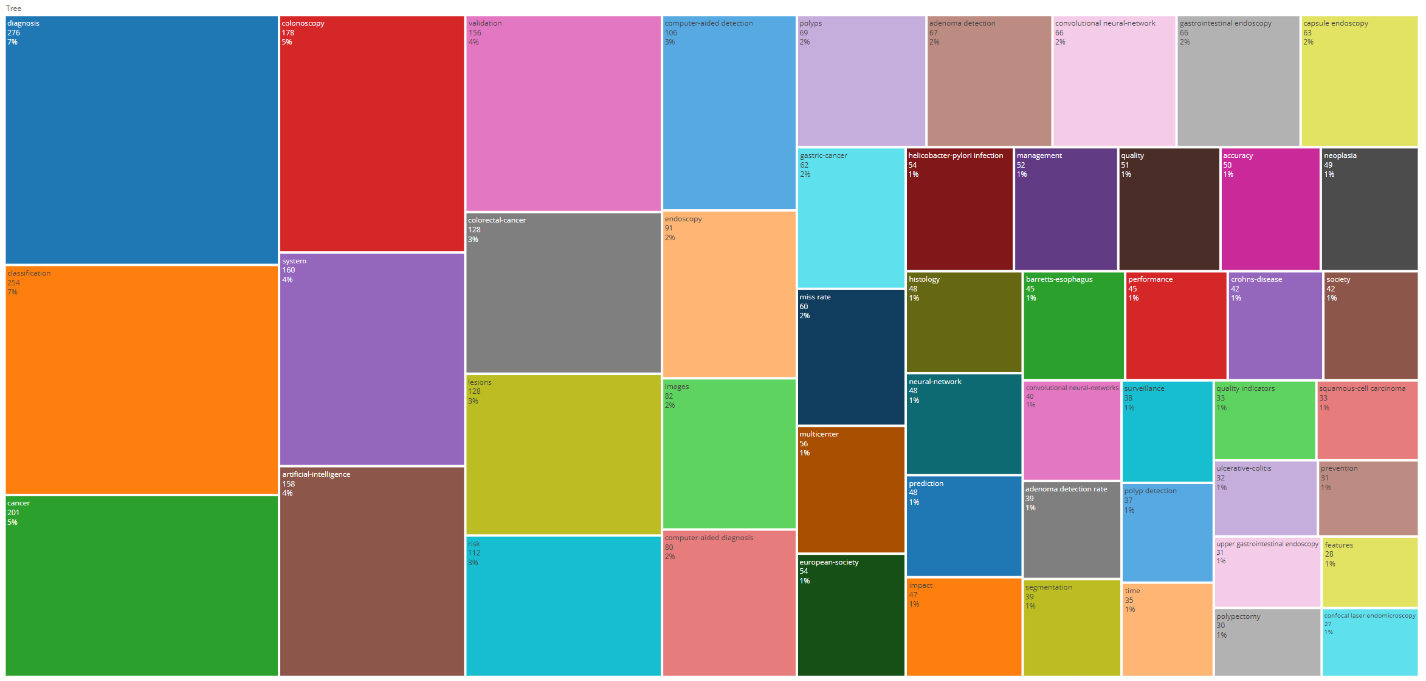


**Figure S2**: Tree map of the keywords in the field of artificial intelligence in endoscopy and colonoscopy


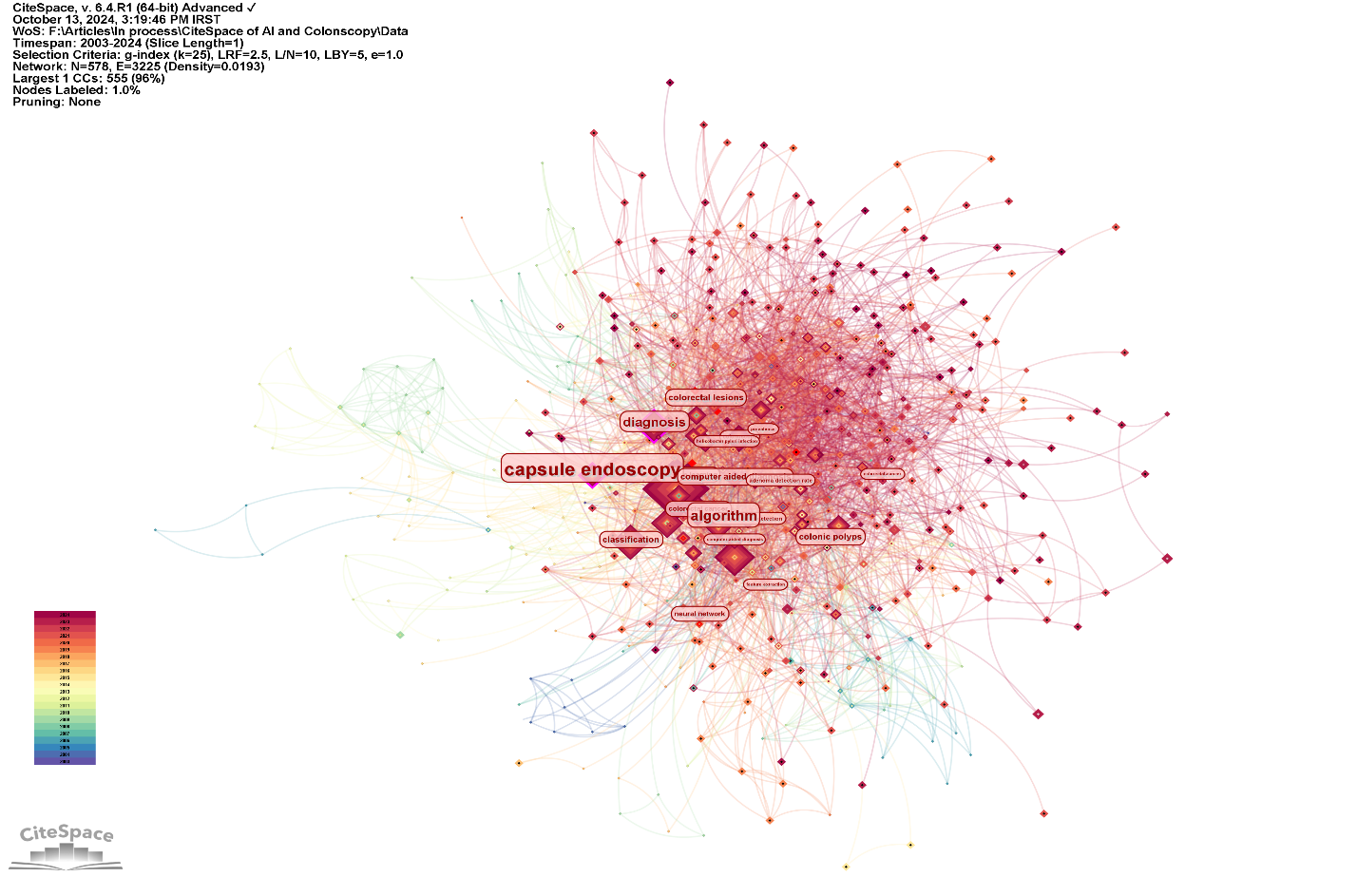


**Figure S3**: Keywords with high centrality in the field of artificial intelligence in endoscopy and colonoscopy


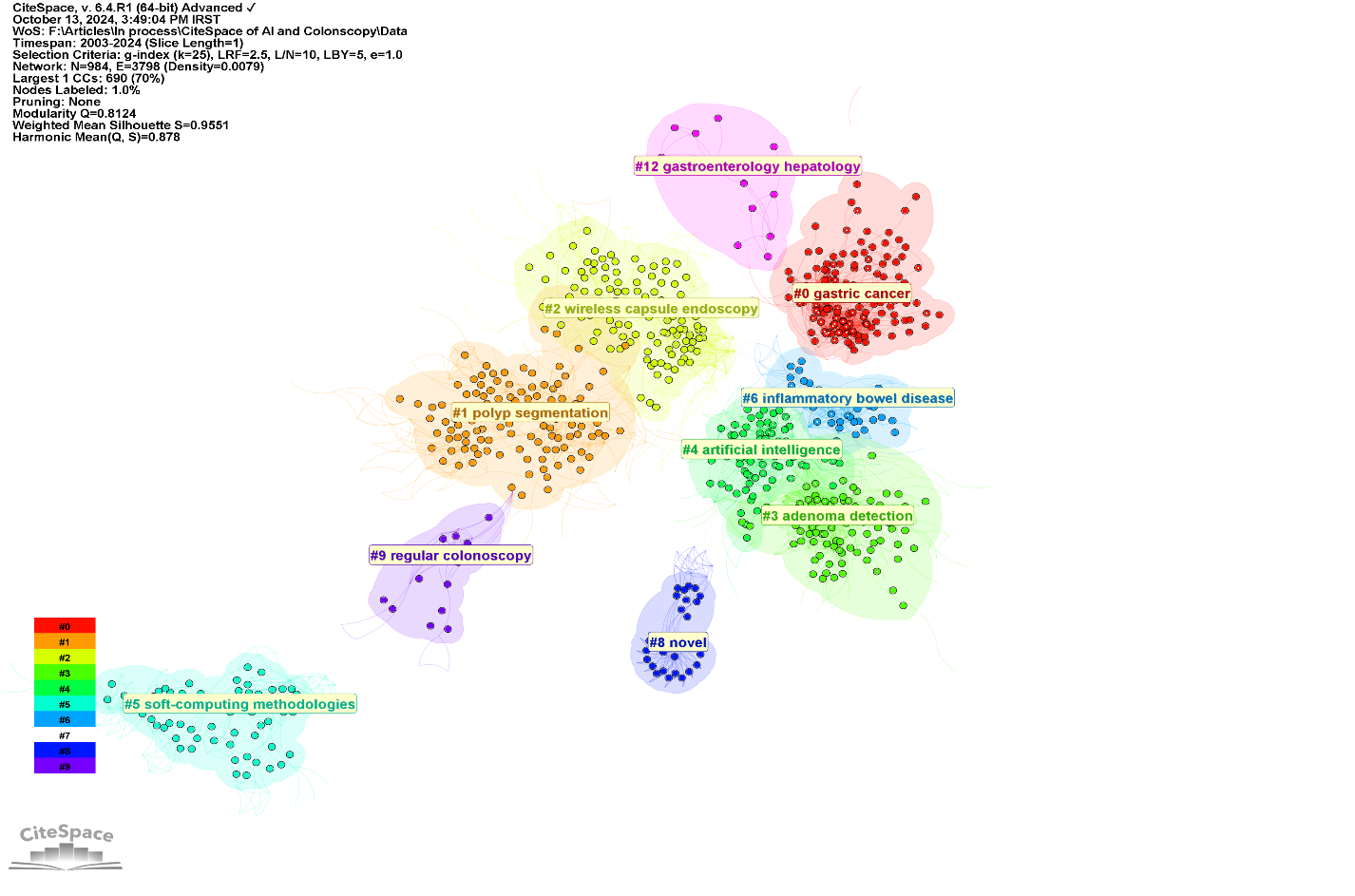


**Figure S4**: Main clusters in the field of artificial intelligence in endoscopy and colonoscopy


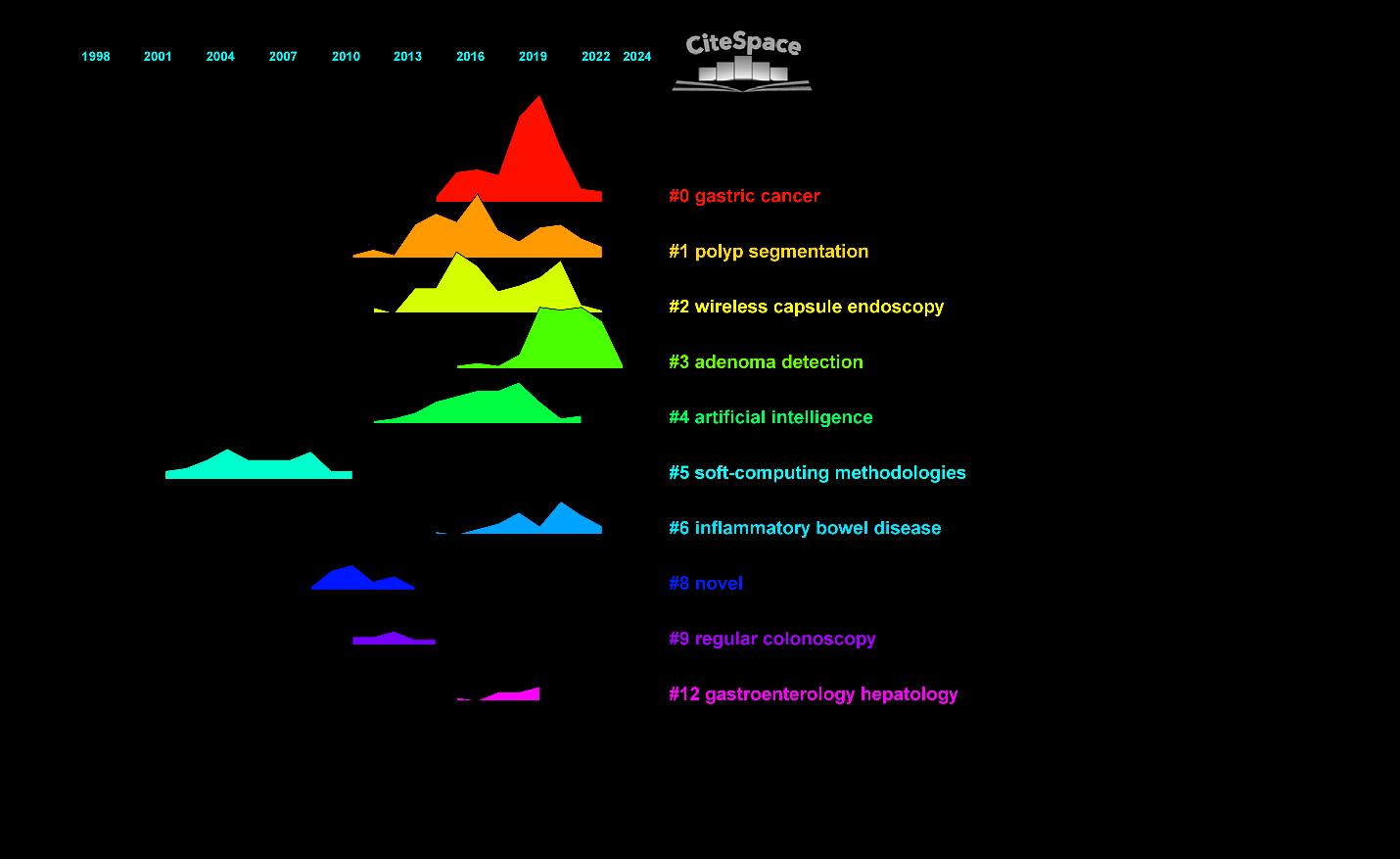


**Figure S5:** Time trend analysis of the main clusters in the field of artificial intelligence in endoscopy and colonoscopy
